# Advanced Stimuli-Responsive Structure Based on 4D Aerogel and Covalent Organic Frameworks Composite for Rapid Reduction in Tetracycline Pollution

**DOI:** 10.3390/molecules28145505

**Published:** 2023-07-19

**Authors:** Wenxin Wang, Wenjing Wang, Ying Liang, Liwen Du, Huan Yang, Haoxiang Ma, Huiting Cheng, Yaqian Yan, Yijun Shen, Qi Chen

**Affiliations:** 1 State Key Laboratory of Marine Resource Utilization in South China Sea, Collaborative Innovation Center of Marine Science and Technology, Hainan University, Haikou 570228, China; wenxinwang@hainanu.edu.cn (W.W.); wenjwang_k@163.com (W.W.); liangying@hainanu.edu.cn (Y.L.); duliwen_hainu@163.com (L.D.); chenghuiting2022@163.com (H.C.); yanyaqian_hainu@163.com (Y.Y.); 2 School of Materials Science and Engineering, Hainan University, Haikou 570228, China; huanhuanyang@hainanu.edu.cn; 3 Deep Sea Engineering Division, Institute of Deep Sea Science and Engineering, Chinese Academy of Sciences, Sanya 572000, China; mahx@idsse.ac.cn

**Keywords:** COFs, stimuli-responsive, photocatalytic, shape memory, 4D aerogel

## Abstract

Intelligentization of materials and structures is an important trend. Herein, the stimuli-responsive 4D aerogel is used as a smart substrate for rapid reduction in tetracycline (TC) pollution, in which this smart stimuli-responsive substrate is designated as P4D. Its fourth dimension originates from stimuli-responsive characteristics with time evolution. Meanwhile, the covalent organic frameworks (COFs) composite is constructed by BiPO_4_ and triazine-based sp^2^ carbon-conjugated g-C_18_N_3_-COF (COF-1), which is another key aspect of COF-1/BiPO_4_@P4D for rapid photocatalytic degradation regarding TC pollution. This emerging smart structure of COFs@P4D can fix programmable temporary state and recover permanent state under thermal or water stimulus without any complicated equipment. Its performance can be tailored by structure, composition, and function. Compared with traditional powder-form photocatalysts, this stimuli-responsive structure provides attractive advantages, such as high permeable framework, self-adaptivity, flexibly customized functional groups, and fast reduction in TC pollution. The predictable development of COFs@P4D could draw much attention for various promising applications in pollution treatment and sensors.

## 1. Introduction

Quickly reducing antibiotic pollution is still a serious challenge in designing, preparing, and engineering materials and structures. There are obvious differences in performance, energy consumption, and cost regarding various treatment methods. Semiconductor photocatalysis is one of the most promising green technologies in terms of environmental purification [1,2,3]. So far, the development of a photocatalytic system that possesses suitable structure, performance, efficiency, energy consumption, and cost is very desirable. Although some photocatalysts have good photocatalytic degradation ability, their powder form and high raw material cost represent a bottleneck regarding widespread application [4,5]. Therefore, new strategies are still highly anticipated to break through that bottleneck.

In order to solve this problem, an original strategy is proposed here that merges conventional photocatalysts into a stimuli-responsive substrate. This work constructs the COF-1/BiPO_4_@P4D composite for demonstrating this strategy. BiPO_4_ is a stable, green, and good ultraviolet-photocatalytic nanomaterial, but the visible light absorption of BiPO_4_ is poor, leading to low utilization of sunlight [6,7]. Modification of BiPO_4_ helps to enhance the visible light absorption and achieve optimization of photocatalytic performance [8,9,10,11,12,13,14]. Combined with a suitable semiconductor, it is a common and effective way to improve the photocatalytic performance of BiPO_4_ [10,11,12].

Porous organic polymers are types of two-dimensional or three-dimensional network skeleton semiconductor materials that are connected by covalent bonds [15]. As porous organic semiconductor materials, COFs have good visible light absorption, designable porous structure, energy band adjustability, and easy modification, resulting in an excellent functional customization material [16,17]. In 2005, Omar M. Yaghi first reported two-dimensional COFs, which initiated the study of COFs [16]. In 2007, Omar M. Yaghi reported three-dimensional COFs for the first time [18]. This marks the beginning of an innovation in the dimension of COFs. Lan et al. synthesized several COF–semiconductor Z-scheme photocatalysts using TiO_2_, BiWO_6_, α-Fe_2_O_3_, and COF-318/316-semiconductor [19]. These COF composites are Z-type heterojunction catalysts connected by covalent bonds. Z-type heterojunction catalysts improve the separation efficiency of photo-generated carriers so as to improve their photocatalytic activity [19].

For overcoming the shortcomings of conventional powder-form photocatalysts, the stimuli-responsive 4D aerogel is used as a smart substrate for developing a COFs@P4D. This stimuli-responsive 4D aerogel is a particularly distinctive and useful smart porous material, and it has fixable temporary shape, multiple stimuli response, programmable fractal structure, high permeability, easy functionalization, and fast enrichment [20]. Its intelligence is reflected in its stimuli-responsive capacity that can recover its original shape from a pre-programmed temporary “freezing” shape under the right stimulus. This 3D aerogel can change state over time, so it is able to be regarded as 4D material. Herein, the fourth dimension is time.

**Figure 1:**
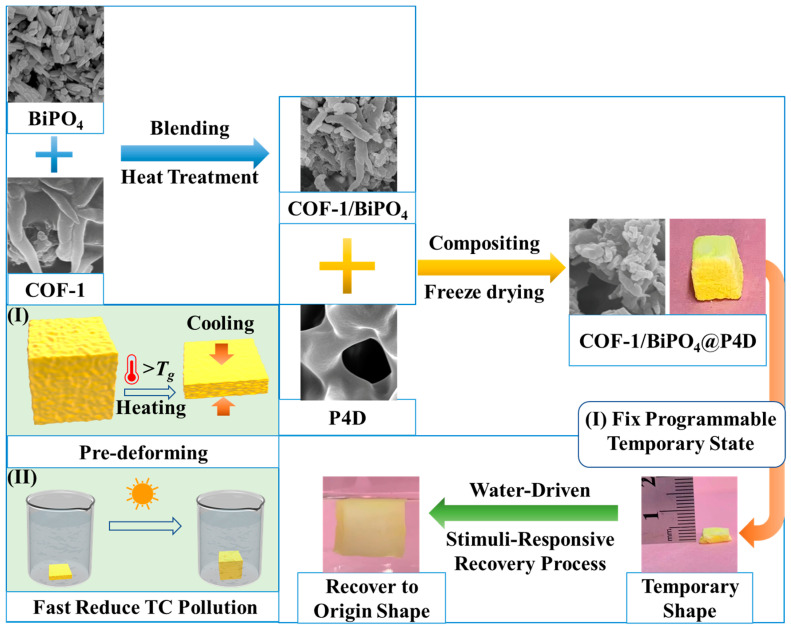
The design strategy of programmable COF-1/BiPO_4_@P4D for fast reduction in TC pollution. (I) Pre-deforming and fixing its programmable temporary shape. (II) The pre-deforming COF-1/BiPO_4_ @P4D for fast reduction in TC pollution process.

The design strategy of an original COFs@P4D is illustrated in Figure 1. The typical stimuli-responsive aerogel substrate is obtained from the precursor system of poly (vinyl alcohol) (PVA) and cross-linker by the precrosslinked ice template strategy based on previous work [20]. Then, the COF-1/BiPO_4_ is coated into P4D. After lyophilization, the COF-1/BiPO_4_@P4D composite is ultimately gained. For comparison with COF-1/BiPO_4_@P4D, the BiPO_4_/P4D and COF-1/P4D composites are prepared by similar preparation processes. The pre-programmed stimuli-responsive process of COF-1/BiPO_4_@P4D consists of programmable fixing and recovering, which distinguishes it from almost all other traditional pollution treatment processes.

The programmable time evolution capacity of P4D is a significant difference and advantage compared to conventional porous substrate. It is well-known that the stimuli-responsive performance could usually be adjusted by its cross-linked molecular network. The submicron structure and functionality of a stimuli-responsive aerogel can be regulated by cross-linker content, precrosslinked parameters, and functional components. Movie S1 and previous work demonstrate the fast enrichment effect of P4D. It is observed that P4D can adsorb crystal violet quickly during the stimuli-responsive recovery process in water. After the extrusion process, the crystal violet can still be firmly absorbed in the P4D [20]. As a common porous substrate, the melamine sponge (MS) only adsorbs crystal violet slowly and slightly in similar environments and at the same time. Remarkably, this stimuli-responsive recovery process of P4D not only accelerates the diffusion-controlled adsorption process but also possesses enrichment capacity of pollutants under the scouring of waste water.

Antibiotics have super stability in a water environment and become one of the most harmful water pollutants in the world. One of the most typical is TC, which accounts for one-third of the total production and consumption of antibiotics and contributes major residues in soil and water around the world [21,22,23,24,25]. Yun Hang Hu verified that TiO_2_ can also degrade 25.1% TC under 700 nm light irradiation [26]. The TC-sensitizing TiO_2_ can absorb visible light, and this surface complex can also generate •O^2−^ to degrade TC. Weidong Shi constructed direct solid-state Z-scheme V_2_O_5_/g-C_3_N_4_ heterojunctions, which have the electrons of high reducibility in the g-C_3_N_4_ conduction band bottom (CBB) and the holes of high oxidizability in the V_2_O_5_ valence band top (VBT) [27]. They can be used to degrade 75.7% TC after 120 min. Xun Cao prepared Bi_2_WO_6_/MOF-ZIF-8 to degrade TC [28]. MOF-ZIF-8 improves the separation of photo-generated carriers and the utilization efficiency of electrons. Herein, the superoxide radicals enhance the photocatalytic degradation ratio of TC. A Z-scheme g-C_3_N_4_/Ag/AgBr heterojunction photocatalyst shows good photocatalytic ability for the degradation of TC [29]. This Z-scheme heterojunction improves the separation efficiency of photo-generated carriers and maintains the oxidation and reduction capability of Ag/AgBr and g-C_3_N_4_, respectively. The results show that •O^2−^ and •OH are the main active species in the photo-degradation of TC. Fang Liu synthesized a S-g-C_3_N_4_/N-TiO_2_@PTFE membrane to degrade TC [30]. The heterojunction between S-g-C_3_N_4_ and N-TiO_2_ can be beneficial for the light absorption and separation of photo-generated carriers, and the superoxide radicals also play an important role in the degradation of TC. As a loading material, PTFE is conducive to recover the S-g-C_3_N_4_/N-TiO_2_ photocatalyst powder. Weiting Yang developed an MS @COF-TpTt composite in which the COF-TpTt grew uniformly on the MS fibers [31]. This composite can not only degrade TC under visible light but also overcomes poor recyclability of the COF powder. It has become clear that the heterojunctions can improve photocatalytic performance, and the loading materials are conducive to recover the photocatalyst powder. Usually, their preparation method is complex and has a high cost. The primary deficiency regarding these loading materials is that they only have carrying capacity. An ideal substrate should possess both carrying capacity and functionality, such as accelerating or enhancing removal capabilities of pollution.

This work, taking TC as an example, tests the pollutant removal of a corresponding sample. The stimuli-responsive 4D aerogel substrate plays a critical role in the rapid adsorption removal of TC pollution during the stimuli-responsive recovery process in water, and COF-1/BiPO_4_ composite works as a key functional component of COF-1/BiPO_4_@P4D for rapid photocatalytic degradation regarding TC pollution. Therefore, this stimuli-responsive structure merges the advantages of the stimuli-responsive 4D aerogel and COFs composites, which has not yet been reported.

The multiscale structure, fundamental properties, photocatalytic performance, and stimuli-responsive performance of smart COFs@P4D are the key elements to influence potential applications, such as pollutant removal.

## 2. Results and Discussion

### 2.1. Fundamental Properties and Microstructure

**Figure 2:**
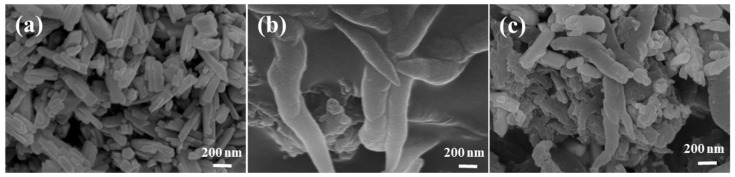
The SEM micrographs of (**a**) BiPO_4_, (**b**) COF-1, and (**c**) COF-1/BiPO_4_.

The morphologies of BiPO_4_, COF-1, and COF-1/BiPO_4_ are studied by scanning electron microscopy (SEM), which can provide the basis for discussing the photocatalysis mechanism of COF-1/BiPO_4_. As shown in Figure 2a, BiPO_4_ is regular nanorod of approximately 100 nm diameter and has good dispersion. These characteristics are conducive to expose more active sites in the photocatalysis process. The SEM image of COF-1 reveals its uniform lance-shaped morphology with about 200 nm diameter and 2 μm length, indicating that it tends to form regular fibrous aggregates at the micro-nano scale (Figure 2b). After several attempts, a blend heat treatment method is used to construct the COF-1/BiPO_4_ composite, maintaining the advantages regarding specific morphology (Figure 2c). In principle, the BiPO_4_ nanorod and the COF-1 fibrous structure closely contact and retain their respective original morphologies in the COF-1/BiPO_4_ composite.

**Figure 3:**
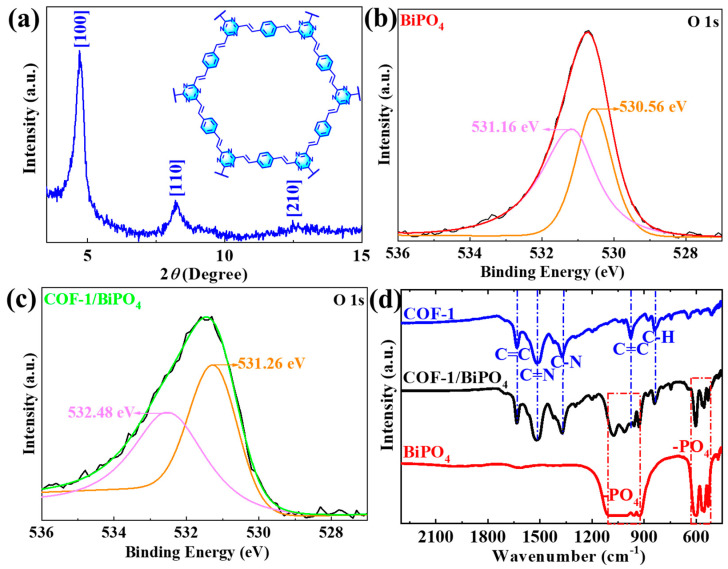
(**a**) XRD pattern of COF-1. The high-resolution XPS spectra of the O 1s regions for (**b**) BiPO_4_ and (**c**) COF-1/BiPO_4_. (**d**) FTIR spectra of BiPO_4_, COF-1, and COF-1/BiPO_4_.

The chemical properties of the COF-1/BiPO_4_ composite are characterized by powder X-ray diffraction (XRD), X-ray photoelectron spectroscopy (XPS), and Fourier transform infrared spectroscopy (FTIR). Figure 3a shows a high-intensity diffraction peak at 4.7, corresponding to the [100] crystal plane. The [110] and [210] crystal plane diffraction peaks are located at 8.2 and 12.7, respectively. The XRD pattern of COF-1 is consistent with those reported in the literature [30]. This indicates that COF-1 is synthesized successfully with good crystallinity. In Figure 3b, there are the lattice oxygen and the adsorbed oxygen of BiPO_4_ at 531.16 eV and 530.56 eV, respectively [10,14]. In the high-resolution XPS spectra of the O 1s regions for COF-1/BiPO_4_, the peaks shift to higher binding energy (Figure 3c). This suggests that there are chemical forces between COF-1 and BiPO_4_ in COF-1/BiPO_4_. Figure 3d includes the FTIR spectra of BiPO_4_, COF-1, and COF-1/BiPO_4_. The absorption bands at 1105 cm^−1^–922 cm^−1^, and 628 cm^−1^–515 cm^−1^ refer to the –PO_4_ of BiPO_4_. The absorption peaks of COF-1 at 1629 cm^−1^ and 833 cm^−1^ refer to the C=C and the C-H of the benzene ring skeleton, respectively. In addition, the absorption peaks of COF-1 at 1517 cm^−1^ and 1361 cm^−1^ refer to the C=N and the C-N of triazine structure, respectively. The absorption peak at 973 cm^−1^ refers to the C=C, which connects the benzene ring to the triazine structure in COF-1. Compared with COF-1 and BiPO_4_, there are the absorption peaks of benzene ring skeleton and triazine structure, as well as the absorption bands of the –PO_4_ in the COF-1/BiPO_4_ composite. This shows that the COF-1/BiPO_4_ composite is synthesized successfully. The changes in these characteristic absorption peaks also show that there are certain bonding interactions between COF-1 and BiPO_4_ in COF-1/BiPO_4_, which also corresponds to the analysis of XPS spectra, as confirmed by Figure 3.

### 2.2. Photocatalytic Performance and Mechanism

**Figure 4:**
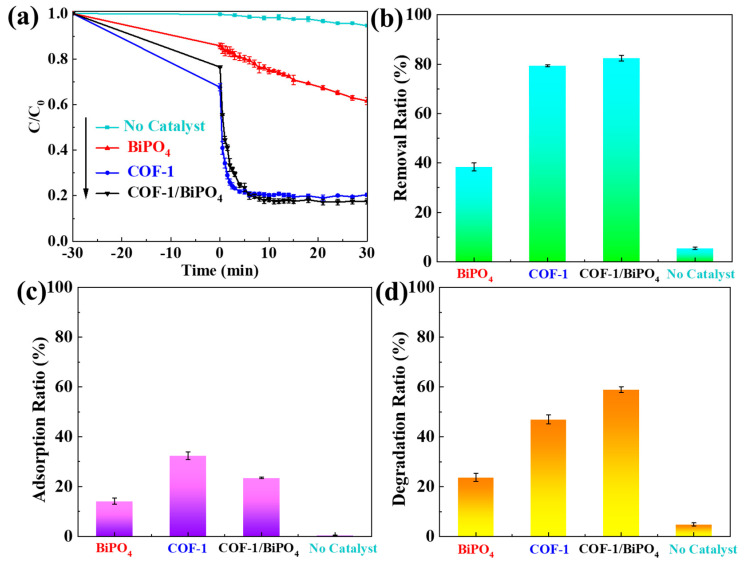
(**a**) BiPO_4_, COF-1, COF-1/BiPO_4_, no catalyst to reduce TC pollution ratio curve. (**b**) Removal ratio of TC in 60 min: BiPO_4_, COF-1, COF-1/BiPO_4_, no catalyst. (**c**) Adsorption ratio of TC in 30 min: BiPO_4_, COF-1, COF-1/BiPO_4_, no catalyst. (**d**) Degradation ratio of TC in 60 min: BiPO_4_, COF-1, COF-1/BiPO_4_, no catalyst.

As a typical antibiotic pollution, TC is used to evaluate the photocatalytic performance of BiPO_4_, COF-1, and COF-1/BiPO_4_. In a TC solution, the samples are evenly dispersed to reach adsorption equilibrium under complete darkness. As shown in Figure 4, the adsorption capacity values of BiPO_4_ and COF-1/BiPO_4_ are 14.2% and 23.5% within 30 min, respectively. COF-1/BiPO_4_ has better adsorption capacity just because COF-1 is multi-hole with larger specific surface area and better adsorption capacity. Interestingly enough, COF-1/BiPO_4_ can quickly reduce TC pollution by 60.2% through photocatalytic degradation only in about 5 min. Its removal ratio of TC can reach nearly 83.6% within only 1 h. In comparison, under the same conditions, BiPO_4_ only removes 38.4% TC. The no catalyst group is the control group for researching the effects of light irradiation to TC. This group result shows that there is little influence on TC under light irradiation. It takes into consideration that the adsorption removal ratio and the photocatalytic degradation removal ratio of COF-1 are about 32.4% and 46.9%, respectively. According to the above results, COF-1 is the key component of COF-1/BiPO_4_ for rapid reduction in TC pollution, and there is a good interaction with COF-1 and BiPO_4_. Figure S1 illustrates the liquid chromatograms and mass spectra of photocatalytic degradation of TC in the presence of COF-1/BiPO_4_. At a retention time of 4.12 min, the eluted TC obviously reduced after a 30 min photocatalytic reaction. A liquid chromatograph mass spectrometer (LC–MS) is used to explore the photoproducts from the photocatalytic oxidation of TC. After a 30 min photocatalytic reaction, the mass spectra only detected a tetracycline ion under the current experimental conditions. According to the detected photoproducts and the related literature reports [32,33,34,35,36,37], it can be concluded that TC can be mineralized safely by COF-1/BiPO_4_, which has been reported in Zhang’s study [32].

**Figure 5:**
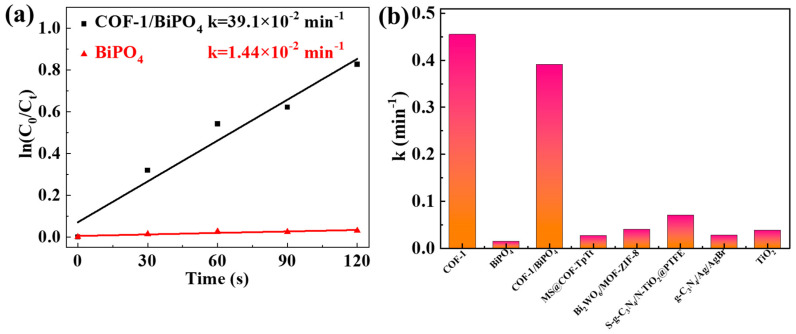
(**a**) The photocatalytic degradation kinetics curve of TC in 120 s: BiPO_4_ and COF-1/BiPO_4_. (**b**) Comparison of TC degradation rate constants in this work and other photocatalysts in the literature [26,28,29,30,31].

In order to better compare the photocatalytic activity of different samples, the Langmuir–Hinshelwood (L-H) equation is used to fit analysis [38]. In Figure 4a, the photocatalytic degradation ratio of the COF-1/BiPO_4_ reaches equilibrium quickly within the first 5 min. Figure 5a is the photocatalytic degradation kinetics curve of TC in 120 s. As expected, COF-1/BiPO_4_ shows a faster degradation rate (0.391 min^−1^) than BiPO_4_ (0.014 min^−1^) with the same conditions. As shown in Figure 5b, the photocatalytic rate of COF-1/BiPO_4_ is much faster than other previously reported photocatalysts, such as COF-TpTt composite (0.026 min^−1^), MOF-ZIF-8 composite (0.040 min^−1^), g-C_3_N_4_ composites (0.070 min^−1^ and 0.028 min^−1^), and TiO_2_ (0.038 min^−1^) [26,38,39,40,41,42,43]. From the foregoing, COF-1 is one of the main influences in improving the photocatalytic activity of COF-1/BiPO_4_. COF-1/BiPO_4_ photocatalysts contain about half the COF-1 levels that COF-1 would have. However, the photocatalytic performance levels of COF-1/BiPO_4_ and COF-1 are similar. This suggests that COF-1 and BiPO_4_ have good synergies, which will be discussed later.

**Figure 6:**
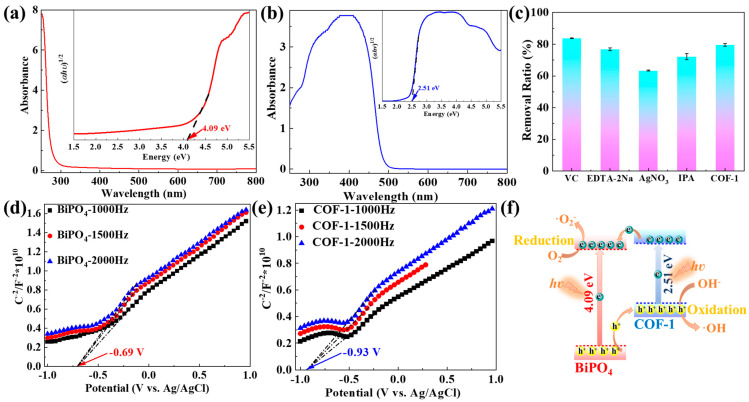
DRS and the band gap calculation of (**a**) BiPO_4_ and (**b**) COF-1. (**c**) Impact of various trapping agents on TC removal ratio: vitamin C (VC) for superoxide anion radical (•O_2_^−^), ethylenediaminetetraacetic acid disodium salt (EDTA-2Na) for hole (h^+^), AgNO_3_ for electron (e^−^), isopropyl alcohol (IPA) for hydroxyl radical (•OH), and COF-1 without trapping agent. Mott–Schottky curves of (**d**) BiPO_4_ and (**e**) COF-1. (**f**) Schematic of photo-generated charge carriers transfer process and photocatalytic mechanism.

In general, the photocatalytic performance is conditioned with migration, separation, and utilization of photo-generated carriers. For a semiconductor photocatalytic reaction, the VBT potential is much more positive the stronger the oxidation capacity, and the CBB potential is much more negative the greater the reduction capacity. The stronger the migration and separation of photo-generated carriers, the better the utilization of photo-generated carriers and more favorable the oxidation–reduction reaction in the photocatalytic process. To obtain further insight into the synergies of COF-1 and BiPO_4_, Figure 6 shows the ultraviolet–visible diffuse reflectance spectra (DRS), the band gap calculation, the impact of various trapping agents, Mott–Schottky curves, and schematic of charge transfer process of BiPO_4_, COF-1, and COF-1/BiPO_4_. The band gap is a very important influence factor on photocatalytic performance. The DRS is used to test the optical property of BiPO_4_ and COF-1. In their absorption spectra, the absorption band edges of BiPO_4_ and COF-1 are about 280 nm and 486 nm, respectively. This shows that the BiPO_4_ is an ultraviolet photocatalyst and the COF-1 displays good visible light absorption capacity. This is one of the reasons that the COF-1 has better light harvesting and photocatalytic activity under simulated solar irradiation (Figure 4 and Figure 5). In addition, the band gaps of BiPO_4_ and COF-1 are calculated to be 4.09 eV and 2.51 eV from the DRS via the Tauc plot, respectively [30,40,41].

In Figure 6c, the trapping experiments of COF-1 provide a basis for further investigating the photocatalytic mechanism. The trapping agents include vitamin C (VC) for the superoxide anion radical (•O_2_^−^), ethylenediaminetetraacetic acid disodium salt (EDTA-2Na) for the hole (h^+^), AgNO_3_ for the electron (e^−^), and isopropyl alcohol (IPA) for the hydroxyl radical (•OH). The removal ration of TC is inhibited after adding EDTA-2Na, AgNO_3_, and IPA. It can be inferred that the photo-generated electrons and holes as well as the generated •OH radicals would benefit from the removal of TC. Figure 6d,e show the Mott–Schottky curves of BiPO_4_ and COF-1 measured under 1000 Hz, 1500 Hz, and 2000 Hz frequency, respectively. The CBB potential values of BiPO_4_ and COF-1 are calculated to be −0.69 V and −0.93 V, respectively.

Based on the above results, a proposed schematic of the photo-generated charge carriers transfer process and photocatalytic mechanism is shown in Figure 6f. When COF-1/BiPO_4_ is illuminated by simulated sunlight, the electrons on the valence band (VB) of BiPO_4_ and COF-1 can be excited to the conduction band (CB), leaving the holes on their VB. The holes on the valence band of BiPO_4_ can transfer to the valence band of COF-1, while the electrons on the conduction band of COF-1 can transfer to the conduction band of BiPO_4_. From the thermodynamic requirement, the holes on the VB oxidize OH^−^ to generate **•**OH and the electrons on the CB reduce O_2_ to yield **•**O_2_^−^. Hence, the photo-generated carriers of COF-1/BiPO_4_ can be more effectively separated than BiPO_4_, which is beneficial to improve its photocatalytic performance due to the effective separation of photo-generated charge carriers improving its utilization intrinsically. Both the photo-generated carriers and the obtained radicals would play important roles in the advanced removal of TC. As a result, COF-1/BiPO_4_ displays higher photocatalytic activity than BiPO_4_. Figure 4, Figure 5 and Figure 6 also indicate that the kinetic requirement may have a more important effect for COF-1/BiPO_4_.

### 2.3. Stimuli-Responsive Photocatalytic Structure

Generally, the photocatalysts are usually in powder form, whose photocatalytic efficiency is intrinsically limited by mass transfer of diffusion. Considering that photocatalysts in powder form are not easy to use and recycle, this stimuli-responsive photocatalytic structure uses a stimuli-responsive 4D aerogel substrate with creativity. Movies S2 and S3 substantiate the water-driven stimuli-responsive capacity of P4D and COF-1/BiPO_4_@P4D. In water, COF-1/BiPO_4_@P4D can recover its original shape within only 10 s at room temperature, which is much faster than most previous water-driven stimuli-responsive polymers in the literature. Its cross-linked network influences dramatically its stimuli-responsive performance [42,43,44]. The water-driven stimuli-responsive behavior of P4D is triggered by the plasticizing effect of water, often with an increase in flexibility of its macromolecule chains [45]. The water-driven stimuli-responsive recovery process of P4D and COF-1/BiPO_4_@P4D can produce suction for facilitating obvious molecules’ and ions’ diffusion to their inner active sites. They have a fast adsorption-enrichment effect during the stimuli-responsive recovery process. This stimuli-responsive 4D aerogel substrate could provide suitable mass transfer pathways.

**Figure 7:**
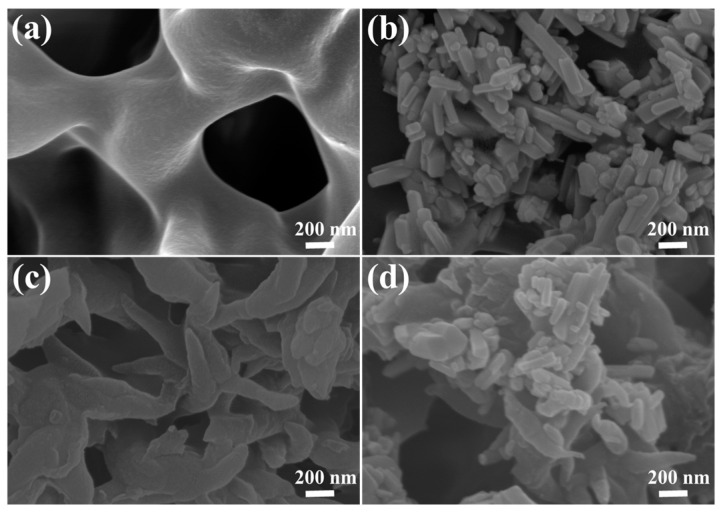
The SEM micrographs of (**a**) P4D, (**b**) BiPO_4_/P4D, (**c**) COF-1/P4D, and (**d**) COF-1/BiPO_4_@P4D.

The diffusion speed of water and the hydrogen bond of the cross-linked network are basic conditions for impacting water-driven stimuli-responsive and adsorption removal performance. As shown in Figure 7, the P4D composites have highly permeable 3D frameworks. The BiPO_4_ of regular nanorod and uniform lance-shaped COF-1 are observed in the SEM micrographs of BiPO_4_/P4D, COF-1/P4D, and COF-1/BiPO_4_@P4D. This shows that BiPO_4_, COF-1, and COF-1/BiPO_4_ dispersed and composited well on the 3D framework surface of P4D. Those nano-functional materials help to raise the specific surface area of the P4D composites, especially for COF-1/BiPO_4_, which is more favorable for increasing the specific surface area and the active site to the benefit of adsorption and photocatalytic removal. The P4D, the BiPO_4_ nanorod, and the COF-1 fibrous structure not only maintain their respective original morphology but also contact closely. The structural characterizations of the P4D composites imply that the interconnected 3D network structure of P4D and large specific surface area of BiPO_4_, COF-1, and COF-1/BiPO_4_ would have beneficial effects on liquid spread, physical adsorption removal, and chemical interaction. The COF-1/BiPO_4_@P4D composite maintains the specific morphology advantages of P4D, BiPO_4_, and COF-1, which would have the benefit of adsorption removal (Movie S1) and photocatalytic removal.

**Figure 8:**
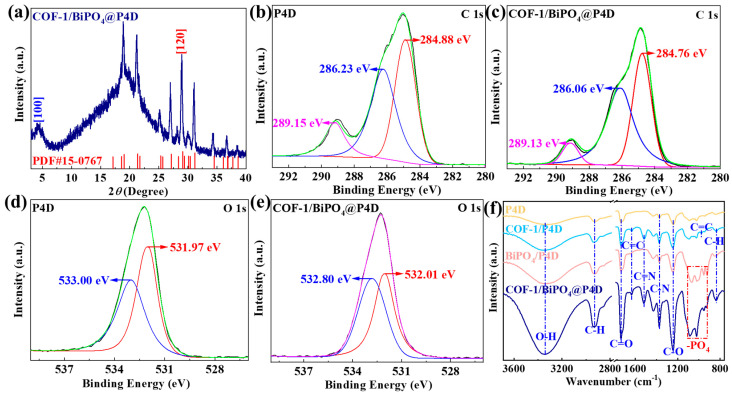
(**a**) XRD pattern of COF-1/BiPO_4_@P4D. The high-resolution XPS spectra of the C 1s regions for (**b**) P4D and (**c**) COF-1/BiPO_4_@P4D. The high-resolution XPS spectra of the O 1s regions for (**d**) P4D and (**e**) COF-1/BiPO_4_@P4D. (**f**) FTIR spectra of P4D, COF-1/P4D, BiPO_4_/P4D, and COF-1/BiPO_4_@P4D.

In Figure 8a, the XRD of COF-1/BiPO_4_@P4D shows diffraction peaks at 4.7, 29.1, and 19.3, corresponding to the [100] crystal plane of COF-1 and the [120] crystal plane of BiPO_4_ and the characteristic peak of P4D, respectively. As shown in Figure 8b, there are C-O and C=O of P4D at 286.23 eV and 289.15 eV, respectively. The 531.97 eV peak is C=O of P4D and the 533.00 eV peak is O-H of P4D (Figure 8c). Compared with P4D, the high-resolution XPS spectra of the C 1s and O 1s regions for COF-1/BiPO_4_@P4D have noticeable changes in binding energy peaks (Figure 8d,e). Figure 8f includes the FTIR spectra of P4D, COF-1/P4D, BiPO_4_/P4D, and COF-1/BiPO_4_@P4D. The absorption bands at 1108 cm^−1^–920 cm^−1^ refer to the –PO_4_ of BiPO_4_/P4D and COF-1/BiPO_4_@P4D. The absorption peaks of COF-1/P4D and COF-1/BiPO_4_@P4D at 1513 cm^−1^ and 1370 cm^−1^ refer to the C=N and the C-N of triazine structure, respectively. It is observed that COF-1/BiPO_4_@P4D has the characteristic peaks of the –PO_4_ and triazine structure. Their FTIR results are consistent with XRD and XPS. They indicate that COF-1/BiPO_4_@P4D is successfully constructed and there are chemical forces between COF-1/BiPO_4_ and P4D.

**Figure 9:**
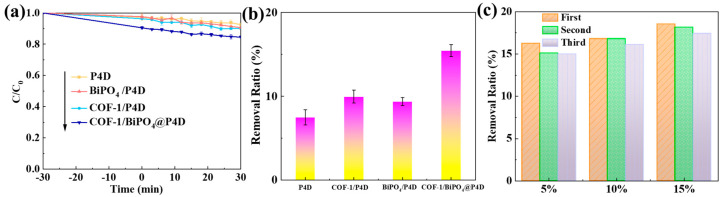
(**a**) P4D, BiPO_4_/P4D, COF-1/P4D, and COF-1/BiPO_4_@P4D to reduce TC pollution ratio curve. (**b**) Removal ratio of TC in 60 min: P4D, COF-1/P4D, BiPO_4_/P4D, and COF-1/BiPO_4_@P4D. (**c**) Removal ratio of TC was repetitively tested in 60 min: 5% COF-1/BiPO4@P4D, 10% COF-1/BiPO4@P4D, and 15% COF-1/BiPO4@P4D.

COF-1/BiPO_4_@P4D has the best removal ratio of TC compared with P4D, BiPO_4_/P4D, and COF-1/P4D (Figure 9a). After 1 h, the TC pollution removal ratio of COF-1/BiPO_4_@P4D is 6.1% higher than BiPO_4_/P4D (Figure 9b). The results in Figure 9c indicate that, under the same conditions, the TC removal ratio of COF-1/BiPO_4_@P4D increases with COF-1/BiPO_4_ content increasing. In addition, the photocatalytic removal ratios of COF-1/BiPO_4_@P4D show good repeatability. As shown in Figure S2, the adsorption isotherm of COF-1 presented a type I adsorption isotherm, so it is a microporous material. Furthermore, its Brunauer–Emmett–Teller (BET) specific surface area is 540 m^2^/g. The temperature dependence of the dynamic compression tests of P4D is shown in Figure S3a. Its shape memory fixing ratio (Rf) and shape memory recovering ratio (Rr) are above 90%. Those results further prove its programmable and reversible transitions between permanent and temporary states. Figure S3b shows that P4D has good thermostability within approximately 230 °C. These features enable COF-1/BiPO_4_@P4D to exhibit water-induced and thermal-induced shape memory performance, as shown in Movie S3 and S4 and Figure S4. It can rapidly recover to its original shape under heat control. In Figure S5a, COF-1/BiPO_4_ shows good visible light absorption, which is consistent with the optical property analysis (Figure 6). The absorption band edges of COF-1/BiPO_4_ and COF-1/BiPO_4_@P4D are about 501 nm and 467 nm, respectively. This indicates that COF-1/BiPO_4_ and COF-1/BiPO_4_@P4D display good visible light absorption capacity. P4D has almost no ability to absorb visible light. The visible light harvesting activity of COF-1/BiPO_4_@P4D is mainly attributed to COF-1/BiPO_4_. The results of Figure S6 show that the contact angles of P4D and COF-1/BiPO_4_@P4D are almost 0^o^, so they have good hydrophilic properties. The water drops are completely wetting and spreading on their mat surface (contact time within 2.8 s and 1.7 s, respectively). It is obvious that COF-1/BiPO_4_@P4D has a better hydrophilic property. These are favorable factors for photocatalytic removal of TC. The P4D of the pre-fixed temporary compressed shape can quickly reach adsorption equilibrium only in 3 min, while the P4D of the original shape must take more than 30 min to reach adsorption equilibrium (Figure S7). The highly permeable 3D framework as well as abundant surface-oxygen-containing, nitrogenous-containing, and phosphorus-containing groups of COF-1/BiPO_4_@P4D are conducive to the spread and the plasticizing effect of water, so it has good water-driven stimuli-responsive and adsorption removal performance. The TC pollution removal capacity of COF-1/BiPO_4_@P4D is attributed to the fast photocatalytic degradation TC of COF-1/BiPO_4_ as well as good adsorption removal TC of COF-1/BiPO_4_ and P4D (Figure 4, Figure 5 and Figure 9). These active influence factors are strong supports for fast reduction in TC pollution.

## 3. Materials and Methods

### 3.1. Materials

Bismuth nitrate pentahydrate (Bi(NO_3_)_3_·5H_2_O), monosodium phosphate (NaH_2_PO_4_), potassium hydroxide (KOH), and ethylene glycol were acquired from Shanghai Macklin Biochemical Technology Co., Ltd. (Shanghai, China). 1,4-diformylbenzene (DFB) and 2,4,6-trimethyl-1,3,5-triazine (TMTA) were ordered from Jilin Chinese Academy of Sciences/Yanshen Technology Co., Ltd. (Jilin, China). Sodium hydrogen carbonate (NaHCO_3_), glutaraldehyde (GA), and PVA were purchased from Shanghai Aladdin Biochemical Technology Co., Ltd. (Shanghai, China).

### 3.2. Preparation of the COF-1/BiPO_4_@P4D

1 mmol Bi(NO_3_)_3_·5H_2_O is mixed with 15 mL ethylene glycol and 1 mmol NaH_2_PO_4_ dissolves in 10 mL water. Their mixture is ivory after stirring 1 h. In the experiment of hydrothermal treatment, this mixture is heated to 200 °C for 12 h in the hydrothermal reactor. BiPO_4_ is washed by ethyl alcohol and water, respectively. Then, it is dried at 60 °C for 12 h. COF-1 is prepared based on our previous research results [30]. COF-1/BiPO_4_ is compounded by combining equal parts of COF-1 and BiPO_4_, which is heat-treated at 200 °C under the atmosphere of nitrogen. On the basis of the first stage of our research [18], P4D is prepared by precrosslinked vacuum-freeze-drying method. The P4D is coated with 5 wt.% COF-1/BiPO_4_, and then the COF-1/BiPO_4_@P4D composite is vacuum-freeze-dried. In order to ensure the comparability of the experiment data, BiPO_4_@P4D and COF-1@P4D are prepared by the same method.

### 3.3. Characterization

SEM (Hitachi S-4800, Hitachi, Chiyoda, Japan) is used to observe the microscopic surface morphology of samples. XRD patterns and the electron binding energy of the samples are measured using XRD measurement system (Rigaku SmartLab, Rigaku, Akishima, Japan) and XPS measurement system (Thermo Scientific Escalab 250Xi, Thermo Scientific, Waltham, MA, USA), respectively. FTIR spectra are recorded on the Perkin-Elmer spectrometer (Spectrum 100, Perkin-Elmer, Waltham, MA, USA). LC–MS (Shimadzu LCMS-IT-TOF, Shimadzu, Kyoto, Japan) is used to explore the photoproducts from the photocatalytic oxidation of TC. Ultraviolet–visible diffused reflectance spectroscopy (Ultraviolet–visible DRS, Shimadzu UV-2600i spectrometer, Shimadzu, Japan) is used to calculate the band gap (*E_g_*) of BiPO_4_ and COF-1. Mott–Schottky curves of BiPO_4_ and COF-1 are recorded by the electrochemical workstation (Vantone PGSTAT101, Vantone, Zurich, Switzerland). N_2_ adsorption–desorption isotherms are measured with automated sorption analyzer (Micromeritics ASAP 2460, Micromeritics, Norcross, GA, USA) and the sample is degassed at 110 °C for 10 h before the test. Dynamic mechanical analysis (DMA) is performed by DMA system (TA Instruments Q850, TA Instruments, New Castle, DE, USA). Thermogravimetric analyses (TGA) are carried out with thermogravimetric analyzer (TA Instruments Q500, TA Instruments, New Castle, DE, USA). The surface hydrophilicity is detected by contact angle meter (Powereach JC2000D4, Shanghai Zhongchen Digital Technology Equipment Co., Ltd., Shanghai, China).

### 3.4. Photocatalytic Performance

The photocatalytic performance is evaluated by photocatalytic degradation of TC solution. Typically, 30 mg sample and 150 mL of 5 mg/L TC solution are mixed by a magnetic stirrer. First, it is stirred evenly for testing its adsorption removal property in the dark, and then the photocatalytic performance is tested using 500 W xenon lamp illumination. The concentration changes regarding TC are monitored by measuring the ultraviolet–visible absorption of the suspensions at appropriate intervals. The characteristic absorption peak of TC at 357nm is used to determine its concentration by a UV-2600i spectrophotometer. VC, EDTA-2Na, AgNO_3_, and IPA are used to test impact of various trapping agents on TC removal ratio.

## 4. Conclusions

Based on 4D aerogel and COF-1/BiPO_4_, this advanced stimuli-responsive structure has the capacity of programmable and fixable temporary state, water-driven stimuli-responsive recovery, adsorption, and photocatalytic removal performance, which are the significant advantages contrasted with traditional powder-form photocatalysts. The photogenic carrier separation and utilization of COF-1/BiPO_4_ is better than BiPO_4_ because of the relatively matched band structure of COF-1 and BiPO_4_. The photocatalytic rates of COF-1/BiPO_4_ are increased by about 11 times, especially compared with ordinary g-C_3_N_4_ or TiO_2_, etc. Especially because of its fixable temporary shape and stimuli-responsive recovery, this smart photocatalytic structure can also accelerate reducing TC pollution, with the suction resulting from water-driven stimuli-responsive recovery as well as the interaction between TC and its active sites. Its functionalities and performances can be optimized by regulating the porous structure, composition, pre-programmed stimuli response, and functional groups. This strategy is expected to solve problems that are difficult for practical applications of powder-form photocatalysts and lower raw material costs of pollutant removal. There are vital advantages in practical applications. The development of this COFs@P4D would be further promoted by exploring various high-value applications.

## Data Availability

The data presented in this study are available on request from the corresponding author.

## Supplementary Materials

The following supporting information can be downloaded at: https://www.mdpi.com/article/10.3390/molecules28145505/s1, Figure S1: (a) Liquid chromatograms of photocatalytic degradation of TC in the presence of the COF-1/BiPO_4_ for different photocatalytic reaction times: 0 min and 30 min. Mass spectra at retention time (RT) = 4.12 min for photocatalytic degradation of TC in the presence of the COF-1/BiPO_4_: (b) photocatalytic reaction time 0 min and (c) photocatalytic reaction time 30 min. Figure S2: N_2_ adsorption–desorption isotherms of COF-1; Figure S3: (a) Cyclic thermodynamic compression experiment and (b) TGA curve of P4D; Figure S4: Thermal-induced shape memory process of COF-1/BiPO_4_@P4D; Figure S5: The optical property of (a) the COF-1/BiPO_4_ and (b) COF-1/BiPO_4_@P4D (illustration is DRS of P4D); Figure S6: (a) The contact angle images of P4D at 0 s and 2.8 s after touching with water drop, respectively. (b) The contact angle images of COF-1/BiPO_4_@P4D at 0 s and 1.7 s after touching with water drop, respectively; Figure S7: The P4D of the pre-fixed temporary compressed shape to reduce TC pollution ratio curve; Movie S1: Rapid adsorption dye during stimuli-responsive recovery process in the water at room temperature; Movies S2 and S3: The stimuli-responsive recovery process of P4D and COF-1/BiPO_4_@P4D in the water at room temperature, respectively; Movie S4: Thermal-induced shape memory process of COF-1/BiPO_4_@P4D.
