# Supplementary material for: Advanced Stimuli-Responsive Structure Based on 4D Aerogel and Covalent Organic Frameworks Composite for Rapid Reduction in Tetracycline Pollution
Source: Molecules. 2023 Jul 19;28(14):5505. doi: 10.3390/molecules28145505 (PMC10386521; doi:10.3390/molecules28145505)
Supplement: Supplementary file 1 [file molecules-28-05505-s001.zip › Supplemental figrues.pdf]

Supporting Information

Advanced Stimuli-Responsive Structure Based on 4D Aerogel and Covalent Organic Frameworks Composite for Rapid Reduction in Tetracycline Pollution

Wenxin Wang [1], Wenjing Wang [1], Ying Liang [1], Liwen Du [1], Huan Yang [2], Haoxiang Ma [3], Huiting Cheng [1], Yaqian Yan [1], Yijun Shen [1,*] and Qi Chen [1,*]

[1] State Key Laboratory of Marine Resource Utilization in South China Sea, Collaborative Innovation Center of Marine Science and Technology, Hainan University, Haikou, 570228, China

[2] School of Materials Science and Engineering, Hainan University, Haikou, 570228, China

[3] Deep Sea Engineering Division, Institute of Deep Sea Science and Engineering, Chinese Academy of Sciences, Sanya, 572000, China

[*] Correspondence: sheny2000@hainanu.edu.cn (Y. J. Shen), chenqi@hainanu.edu.cn (Q. Chen)

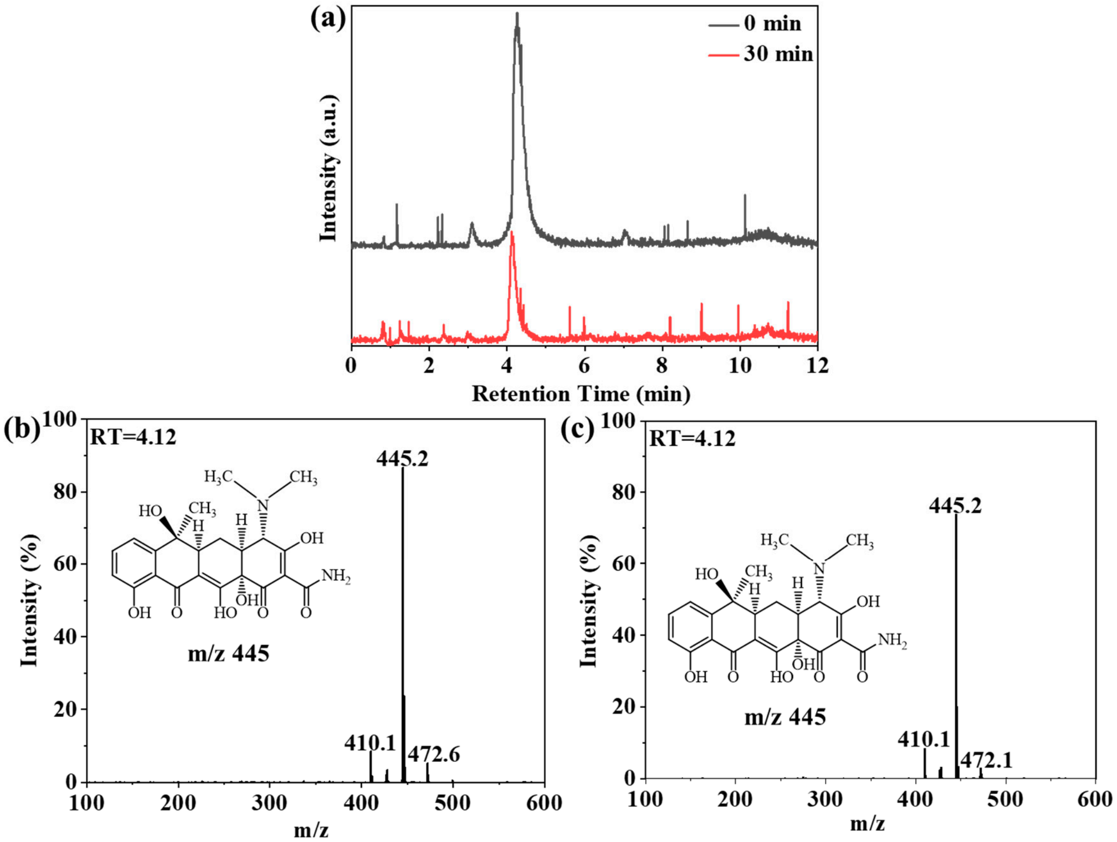

Figure S1. (a) Liquid chromatograms of photo-catalytic degradation of TC in the presence of the COF-1/$BiPO_4$ for different photo-catalytic reaction times: 0 min and 30 min. Mass spectra at retention time (RT) = 4.12 min for photo-catalytic degradation of TC in the presence of the COF-1/$BiPO_4$: (b) photo-catalytic reaction times 0 min and (c) photo-catalytic reaction times 30 min.

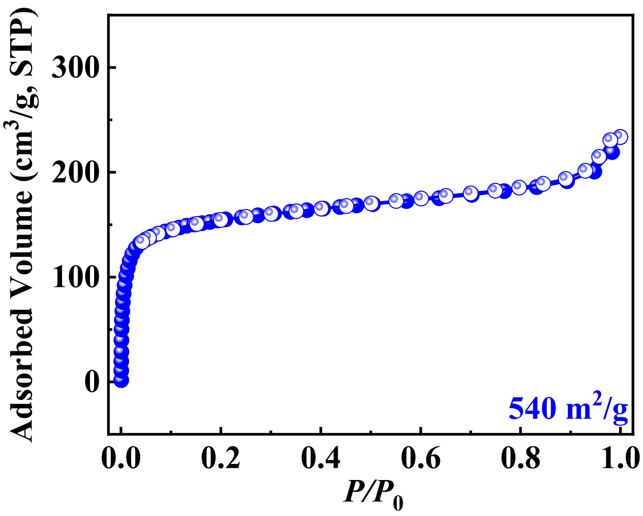

Figure S2. N_2 adsorption-desorption isotherms of COF-1.

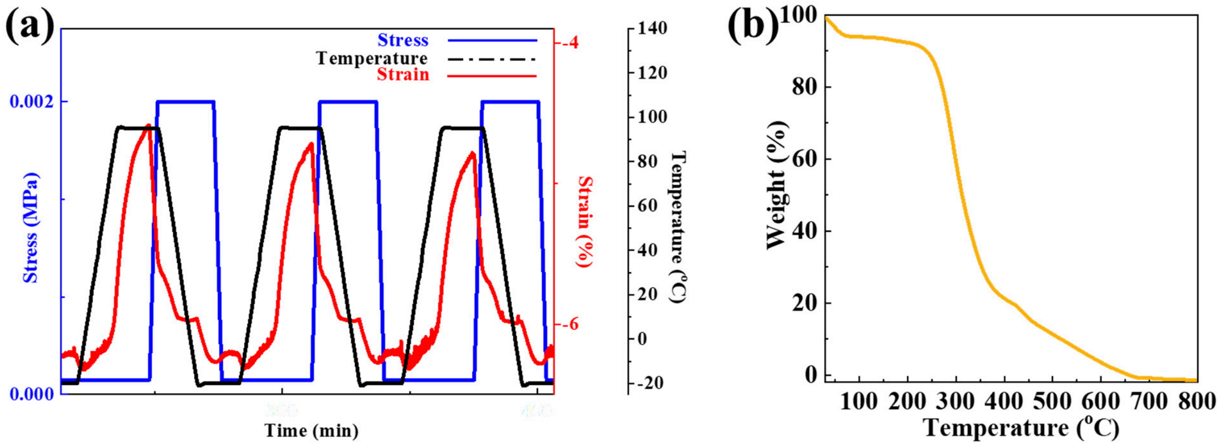

Figure S3. (a) Cyclic thermodynamic compression experiment and (b) TGA curve of P4D.

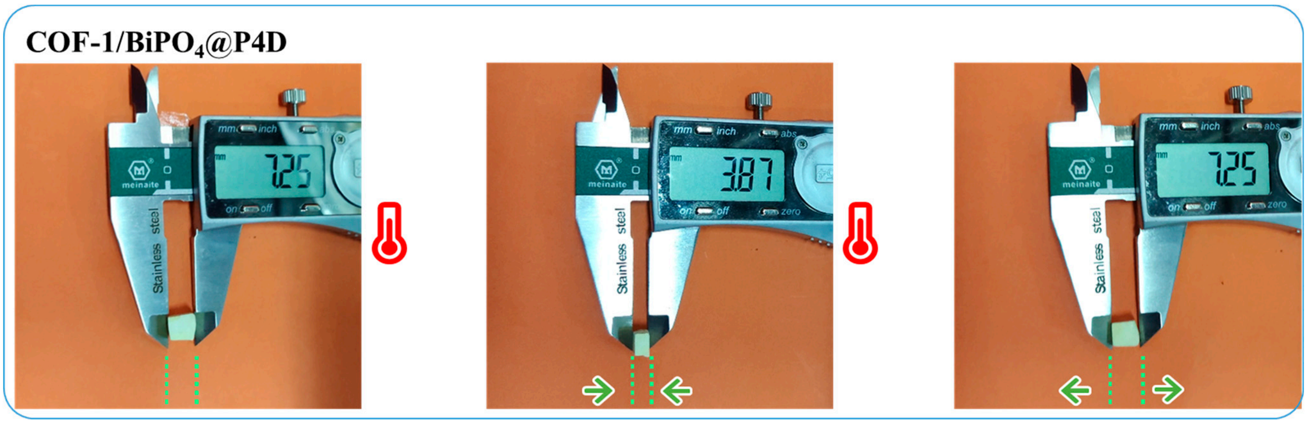

Figure S4. Thermal-induced shape memory process of COF-1/$BiPO_4$@P4D.

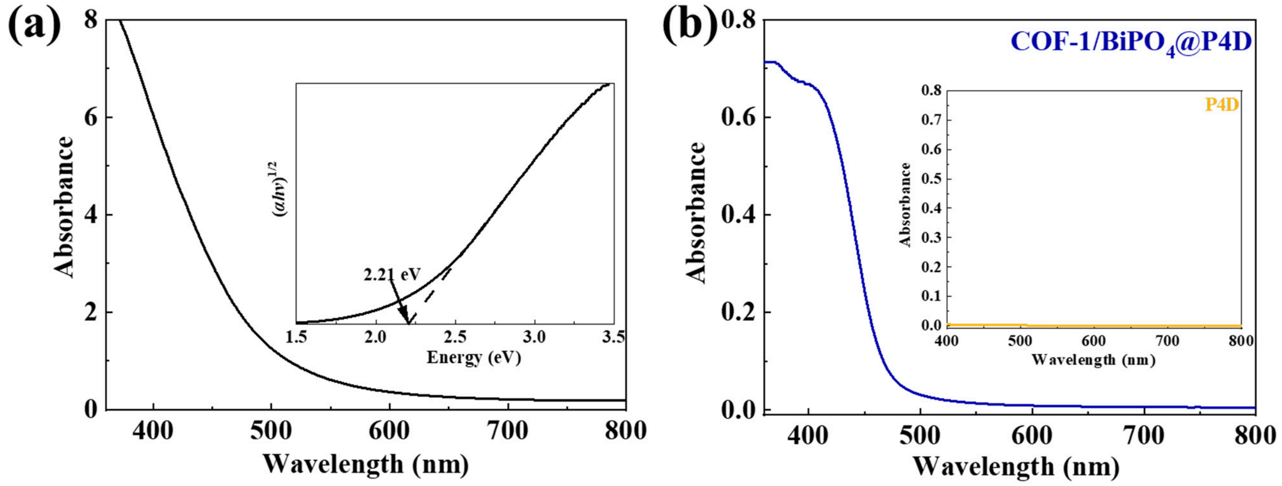

Figure S5. The optical property of (a) the COF-1/$BiPO_4$ and (b) COF-1/$BiPO_4$@P4D (illustration is DRS of P4D).

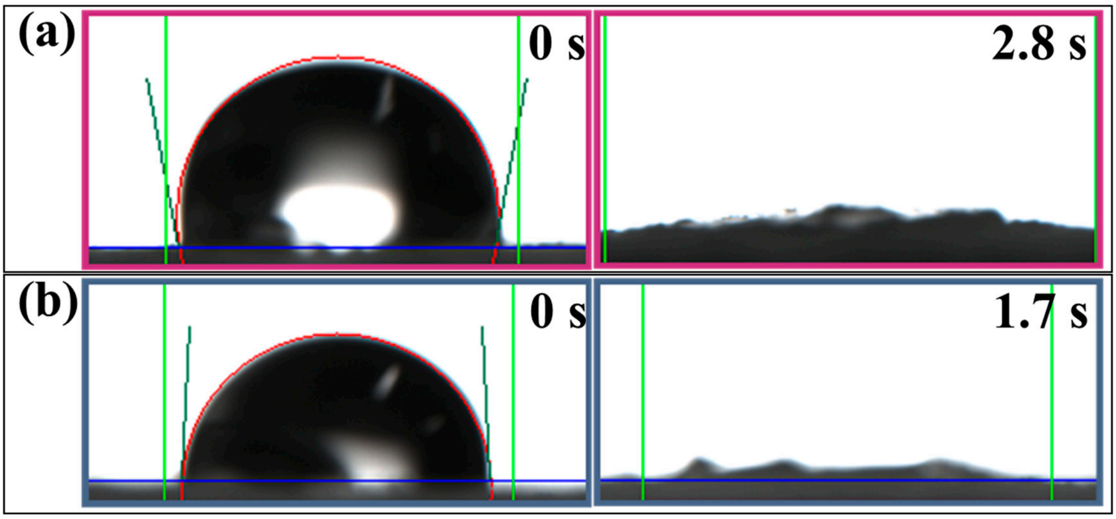

Figure S6. (a) The contact angel images of P4D at 0 s and 2.8 s after touching with water drop, respectively. (b) The contact angel images of COF-1/$BiPO_4$@P4D at 0 s and 1.7 s after touching with water drop, respectively.

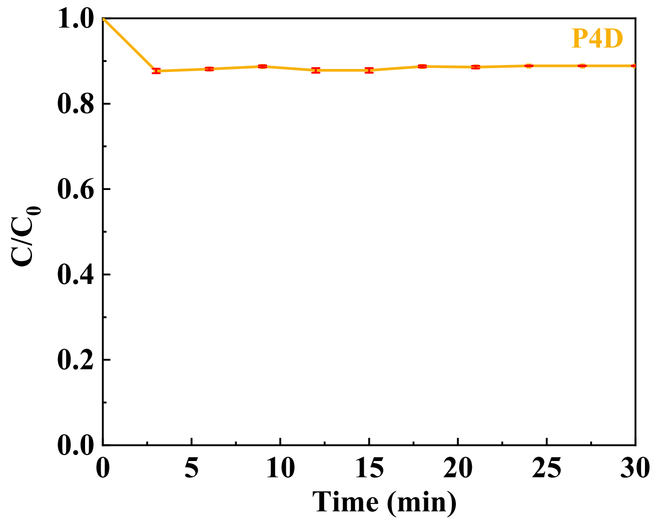

Figure S7. The P4D of the pre-fixed temporary compressed shape to reduce TC pollution ratio curve.
